# Comparison of optical coherence tomography angiography metrics in primary angle-closure glaucoma and normal-tension glaucoma

**DOI:** 10.1038/s41598-021-02296-x

**Published:** 2021-11-30

**Authors:** Ruyue Shen, Yu Meng Wang, Carol Y. Cheung, Poemen P. Chan, Clement C. Tham

**Affiliations:** 1 Department of Ophthalmology and Visual Sciences, The Chinese University of Hong Kong, 4/F Hong Kong Eye Hospital, 147K Argyle Street, Kowloon, Hong Kong SAR The People’s Republic of China; 2 Hong Kong Eye Hospital, Kowloon, Hong Kong SAR The People’s Republic of China

**Keywords:** Glaucoma, Eye diseases

## Abstract

To investigate the peripapillary vascular metrics in early normal tension glaucoma (NTG) and early primary angle closure glaucoma (PACG) eyes using optical coherence tomography angiography (OCT-A). One or both eyes of each subject were imaged for a 3 × 3 mm peripapillary region by swept-source OCT-A (DRI-OCT Triton, Topcon, Japan) and assessed by an automated MATLAB program. OCT-A metrics including circumpapillary vessel density (cpVD) and fractal dimension (cpFD) were compared. Their association with visual field (VF) parameters and retinal nerve fiber layer (RNFL) thickness were determined. Sixty-eight eyes of 51 PACG, 68 eyes of 48 NTG, and 68 eyes of 49 control subjects were cross-sectionally analyzed. NTG eyes had significantly lower global cpVD (52.369 ± 0.781%) compared with PACG eyes (55.389 ± 0.721%, *P* = 0.004) that had comparable disease severity and average RNFL thickness. Multivariable analysis revealed that, for PACG and NTG eyes, decreased cpVD ([PACG] β = −4.242; CI: −8.120, −0.363 vs [NTG] β = −5.531; CI: −9.472, −1.590) and cpFD ([PACG] β = −8.894;CI: −11.925, −5.864 vs [NTG] β = −12.064; CI: −17.095, −6.932) were associated with decreased RNFL thickness (all *P* ≤ 0.032); with a stronger association between decrease cpFD and decreased RNFL thickness in NTG eyes (*P* = 0.028). Decreased cpVD was associated with decrease mean deviation (MD) in NTG eyes (β = −0.707; CI: −1.090, −0.324; *P* ≤ 0.001) and not associated with the visual field parameters in PACG eyes. Early NTG had lower global cpVD compared with early PACG, despite similar disease severity and average RNFL thickness.

## Introduction

Glaucoma is a heterogeneous group of progressive optic neuropathy characterized by degeneration of retinal ganglion cells (RGCs), resulting in a characteristic optic neuropathy and visual field (VF) loss^1^. Some patients experienced continuous VF deterioration despite adequate intraocular pressure (IOP) control, especially in normal tension glaucoma (NTG)^2^. Studies suggested that vascular factors, other than the IOP-dependent risk factors, may be an important pathogenic mechanism of NTG^3–6^. For instance, population-based studies demonstrated the association between retinal vascular changes observed on retinal photographs and glaucoma^7, 8^; NTG eyes was also observed to have significantly narrower retinal arteriolar caliber compared to eyes with primary open angle glaucoma (POAG) and higher IOP (high-tension glaucoma, HTG)^9^. In contrast, primary angle closure glaucoma (PACG) is likely to be more IOP-dependent^10^. If this were the case, one would expect a difference in the pattern of microvascular changes between the two glaucoma subtypes throughout the spectrum of different disease severity.

Optical coherence tomography angiography (OCT-A) allows direct, three dimensional, non-invasive, and depth-resolved visualization of the retinal and choroidal microvasculature through *en face* reconstruction of OCT combined with motion contrast processing^11^. Hence, peripapillary optic disc perfusion and vessel density (VD) can be quantitatively measured. Lower disc flow index and VD have been observed in POAG eyes compared with normal controls^12, 13^; and also correlated with more severe disease^14^. Microvascular reduction was also associated with VF defects in a region-specific manner^15^. These suggested that OCT-A may be potentially useful in detecting retinal microvascular change and vascular mechanism of glaucoma^13, 16^.

It is unclear whether the characteristic pattern of vascular changes, whilst associated with glaucoma, are the cause or consequence of RGCs loss. Vascular impairment per se could deplete the RGCs’ blood supply and lead to their loss; RGCs death per se could also lead to reduced metabolic demand and the consequential vascular reduction. Neither the comparison of glaucoma patients with normal subjects^15^, nor the comparison of eyes with the same glaucoma subtype of different severities^14^, could specifically address this question. Comparing the microvasculature of PACG eyes (a predominantly IOP-dependent glaucoma subtype) and NTG eyes (a predominantly non-IOP-dependent glaucoma subtype) could reveal the role of vascular mechanisms in the disease pathogenesis, especially at the early stage of the disease in which early vascular and retinal nerve fiber layer (RNFL) changes could be observed. The purpose of the present study was to evaluate peripapillary vasculature between early PACG and early NTG, as well as to compare the strength of associations in OCT-A vascular metrics with structural and functional glaucoma parameters in PACG and NTG.

## Methods

### Subjects

This is a cross-sectional clinical study. The study was conducted in accordance with the ethical standards stated in the 2013 Declaration of Helsinki and approved by Hong Kong Kowloon Central Research Ethics Committee with written informed consent obtained. Patients with PACG, NTG, and normal control were recruited from June 2018 to May 2019 at Hong Kong Eye Hospital and the CUHK Eye Centre. The PACG subjects were recruited from an ongoing population-based study, the CUHK PACG Longitudinal (CUPAL) study, which was described in detail previously^17, 18^. One or both eyes of a subject could be enrolled.

### Clinical examinations

A full ophthalmic assessment was carried out. This included measurement of best-corrected visual acuity (BCVA), refractive error with an autorefractor (Nidek ARK-510A, Gamagori, Japan), axial length (AL) as well as anterior chamber depth (ACD) with A-scan biometer (AL-100, Tomey, Nagoya, Japan), IOP with Goldmann applanation tonometry, central cornea thickness (CCT) with non-contact tonopachymeter (TONOPACHY™ 530P, Nidek co., Ltd., Gamagori, Japan), dark room gonioscopy, anterior segment slit-lamp biomicroscopy, dilated fundal examination with assessment of the optic disc (20D and 90D lens), and VF examination by static automated white-on-white threshold perimetry (Humphrey Field Analyzer; 24-2 Swedish interactive threshold algorithm; Carl Zeiss Meditec, Dublin, CA, USA). IOP was measured twice for each eye. If the two readings differed by ≤ 2 mmHg, the mean was recorded as the IOP measurement. Otherwise, a third reading was performed, and the mean was recorded. Gonioscopy was carried out in a dark room. Spherical equivalent (SE) refraction was calculated as the sum of the spherical value and half of the cylindrical value. Peripapillary RNFL thickness was measured with Spectralis SD-OCT (HRA + OCT, Heidelberg Engineering, Heidelberg, Germany). OCT-A imaging was taken by a swept-source OCT (detailed in the session “OCT-A imaging”).

### Definitions

All NTG and PACG eyes had structural and functional evidence of glaucoma, including glaucomatous optic disc cupping, thinning of the RNFL, loss of neuroretinal rim, and minimal criteria for glaucomatous VF defect as per published standard^2^: glaucoma hemifield test result outside normal limits, pattern standard deviation (PSD) with *P* < 0.05 or a cluster of 3 or more points in the pattern deviation plot in a single hemifield with *P* < 0.05, one of which must have *P* < 0.01. Any one of the preceding criteria, if repeatable, was considered sufficient evidence of a glaucomatous VF defect. Additional criteria of NTG eyes were adopted from the Collaborative Normal Tension Glaucoma study^2^ that included: (1) six median untreated IOP readings consistently less than 21 mmHg, with no more than 1 reading equal to 23 or 24 mmHg and no single measurement more than 24 mmHg; at least 2 readings were obtained at a different time of the day from the rest, and (2) open drainage angle (Shaffer grade II or above) on dark room gonioscope. Eyes with PACG had (1) a total of ≥ 180° of angle closure obliterating the trabecular meshwork (synechial or appositional), and (2) untreated IOP of > 21 mmHg^19^. Patients with early glaucoma were recruited in this study according to the modified Hodapp-Anderson-Parrish (HAP) staging^20^—mean deviation (MD) score of −0.01 to −6.00; any point below 5% on the probability plot (with > 3 contiguous, and > 1 of the point below 1%); PSD at *P* < 0.05, or “outside normal limits” for the glaucoma hemifield test. Age-matched normal controls had open anterior chamber angle (Shaffer grade II or above), untreated IOP of < 21 mmHg, no abnormalities in the anterior segment and posterior segment on clinical examination, no structural or functional evidence of glaucoma, and no family history of glaucoma or other ocular diseases except visually insignificant cataract or myopia/hyperopia of less than 3 diopters (D). The latter is a measure to avoid inclusion of patients with extreme AL that may affect the image scale, hence the quantitative metrics as well as area over which measurements were made.

Exclusion criteria included age of < 18 years old, BCVA of worse than 20/40, glaucoma stage of moderate or worse according to the HAP staging^20^, unreliable VF examination (fixation loss > 20%, false positive > 15%, or false negative > 15%, or other evidence of poor quality including inattention fixation, eyelid or lens rim artifacts, fatigue effects, and abnormal results caused by other factors other than glaucoma), OCT images with a signal strength of less than 20, low quality OCT-A imaging (see the session “OCT-A Quality Control”), evidence of secondary causes of glaucoma (e.g. steroid-induced glaucoma, history of uveitis, angle recession resulting from previous trauma), history of ocular surgery except for uneventful cataract surgery and/or laser iridotomy conducted more than 6 months before inclusion, and eyes with other retinal diseases that might affect the measurement of the microvasculature (e.g., diabetic retinopathy, epiretinal membrane, or age-related macular degeneration). We also excluded patients who had acute angle closure attack.

### OCT-A imaging

All study subjects underwent OCT-A using the swept-source OCT (Triton DRI-OCT, Topcon, Tokyo, Japan). Volumetric OCT scans centered on the optic disc were obtained with a scan area of 3 × 3 mm containing 320 × 320 A-scans. The latest built-in software (IMAGEnet6) was used to generate OCT angiograms, which provides improved detection sensitivity of low blood flow and reduced motion artifacts without compromising axial resolution^21^. An OCT-A quality score ranged from 0 to 100 was automatically given by the software for each volumetric OCT scan. The built-in software can separately detect four horizontal depth-resolved segments (the optic nerve head, vitreous, radial peripapillary capillary, and the choroid). The peripapillary capillaries were analyzed from the segment of the optic nerve head, extending from the internal limiting membrane (ILM) to the boundary of RNFL.

### OCT-A quality control

Each OCT-A image and OCT cross-sectional B-scan images were evaluated in the CUHK Ocular Reading Center by a single reader (YMW), who was masked to the diagnosis and clinical demographics of the participants. OCT-A images with poor image quality or significant image artifacts were excluded before the quantitative analysis, including: (1) image quality score less than 40, (2) inaccurate segmentation of tissue layers or labs, (3) motion artifacts (e.g., vessel discontinuity), (4) blurry images, (5) poor centration or (6) signal loss (e.g., due to eye blinking).

### Quantification of retinal microvasculature

**Figure 1:**
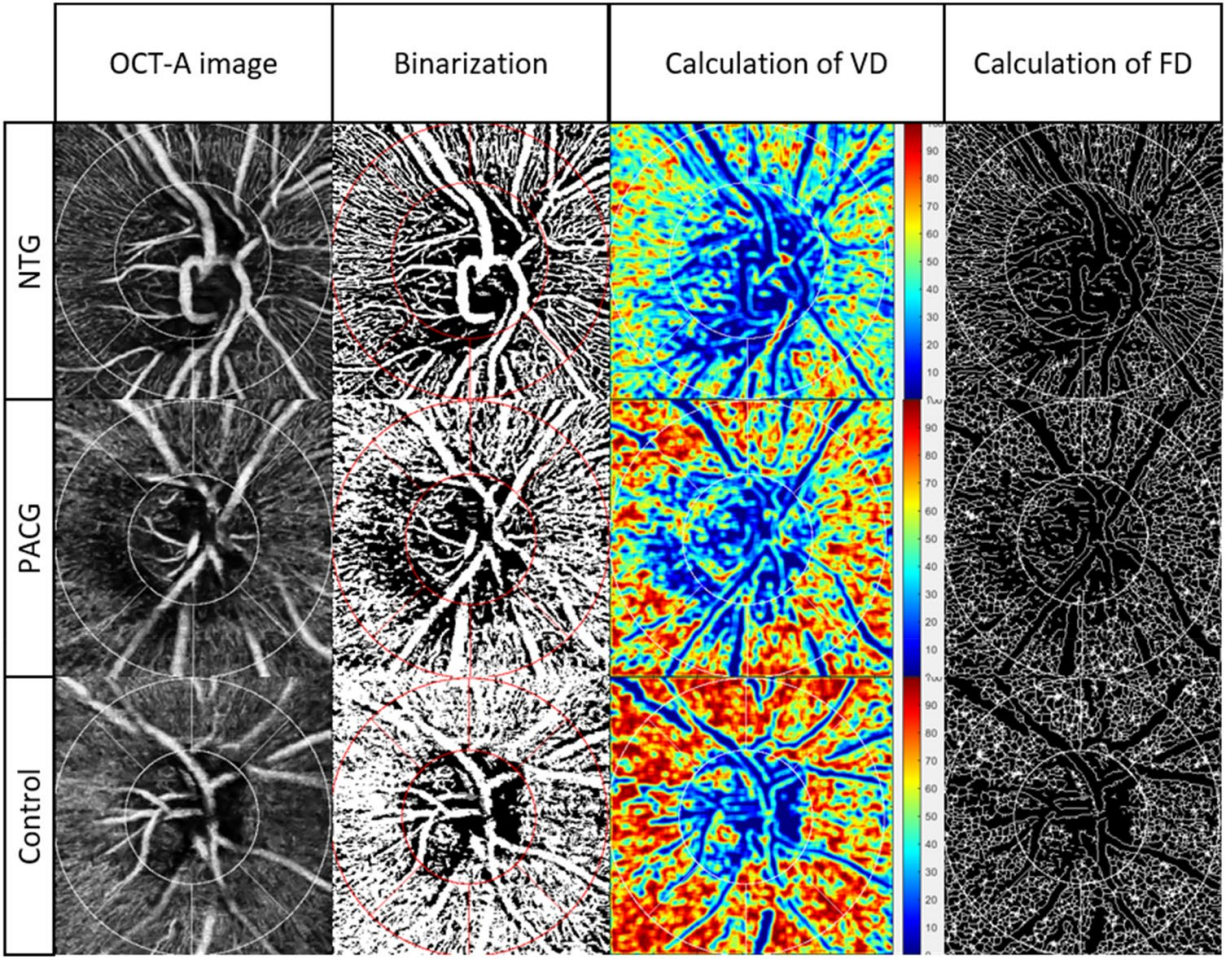
Quantification of peripapillary microvasculature from OCT-A images in NTG, PACG and normal control subjects. A series of OCT-A metrics, including circumpapillary vessel density (cpVD) and fractal dimension (cpFD) were automatically calculated after large retinal vessels removal by our customized MATLAB (MathWorks, Natick, MA) programme.

All included OCT-A images were imported into a customized automated MATLAB program for quantitative analysis. The detailed process of the retinal microvasculature quantification and reliability assessments have been reported before^22^. Quantitative vascular indices—including circumpapillary vessel density (cpVD) and fractal dimension (cpFD)—were obtained after large retinal vessels removal. cpVD was calculated as the percentage of area and was defined as perfusion regions over the total area within the circumpapillary region in the binarized image^23^. cpFD is a mathematical index that quantifies complex geometric patterns in structures that are self-similar in their scaling patterns, and is considered as a classifier to discriminate the class of normal structures from abnormal or pathological structures^24^. It represents a global measurement of the complexity of the vascular branching pattern^25, 26^; a higher cpFD value indicates a more complex vascular branching pattern, whereas a lower cpFD value indicates a sparser vascular network. In our study, the MATLAB program automatically determined cpFD from the skeletonized image using the box-counting method^27^. Figure 1 demonstrates the quantitation of retinal microvasculature from OCT-A images using our customized program in normal, PACG, and NTG eyes. The reliability test showed the values of intraclass correlation coefficient (ICC) were all larger than 0.75 for the OCT-A metrics (Table S1).

### Statistical analysis

All statistical analyses were performed using the IBM SPSS statistics for Windows, Version 25.0 (IBM Corp., Armonk, NY, USA). Normality of the variables was assessed by histogram. A generalized estimating equation (GEE) with Sequential Bonferroni post-hoc test was used to compare the parameters among three groups to adjust the inter-eye correlation. A chi-square test was used to compare the categorical variables among the three groups. Univariable and multivariable linear regression models, using GEE with the exchangeable working correlation structure were performed to determine the associations of average RNFL thickness and visual field parameters for each standard deviation change with OCT-A metrics. Univariate analyses were performed to determine the association of demographic and ocular variables with RNFL thickness and visual field parameters. Variables with *P* value < 0.1 from univariate analyses were selected into multivariable analyses. The regression coefficients in the linear regression models between PACG and NTG group were compared by forming an interaction term^28^. A *P* value of < 0.05 was considered statistically significant.

## Results

Two hundred and eighty-nine eyes (90 eyes of PACG, 115 eyes of NTG, and 84 eyes of normal control) were originally selected in this study. Eighty-five eyes (22 eyes of PACG, 47 eyes of NTG and 16 eyes of normal control) were excluded due to low quality score (5 eyes of PACG, 6 eyes of NTG), motion artifacts (8 eyes of PACG, 22 eyes of NTG, 10 eyes of normal control), blurred images (2 eyes of PACG, 8 eyes of NTG, 2 eyes of normal control), signal loss (4 eyes of PACG, 7 eyes of NTG, 2 eyes of normal control), and poor centration (3 eyes of PACG, 4 eyes of NTG, 2 eyes of normal control), leaving a total of 204 eyes (68 eyes from 51 PACG subjects, 68 eyes from 48 NTG subjects and 68 eyes from 49 normal subjects) for the final analysis.

Table 1 shows the demographics and clinical characteristics of the participants. There were no significant differences in sex, age, and CCT amongst the three groups. The average BCVA was > 0.8 (Snellen scale) for all the participants, with the PACG eyes having the lowest value amongst the groups (*P* < 0.001 versus control and *P* = 0.003 versus NTG eyes). The PACG eyes had shorter ACD, shorter AL, and a higher IOP compared with either NTG eyes (all *P* ≤ 0.001) or normal controls (all *P* ≤ 0.001). Eyes with glaucoma had lower MD, higher PSD, lower VFI, and used more glaucoma medications than the normal controls (all *P* < 0.001), without significant differences between the two groups of glaucoma.

**Table 1:** Demographics and ocular characteristics among PACG, NTG, and Controls.

Variables	PACG	NTG	Controls	*P* value		
				**PACG vs NTG**	**PACG vs Controls**	**NTG vs Controls**
Subjects (n)	51	48	49	N/A	N/A	N/A
Eyes (n)	68	68	68	N/A	N/A	N/A
Sex (female/male)	35/16	27/21	33/16	0.218	0.891	0.277
Age at recruitment (year)	59.82 ± 0.91	62.42 ± 1.30	59.42 ± 1.15	0.100	0.783	0.083
SE (diopter)	0.14 ± 0.22	−1.35 ± 0.32	−1.20 ± 0.27	** < 0.001**	** < 0.001**	0.720
AL (mm)	22.43 ± 0.12	24.60 ± 0.17	24.26 ± 0.15	** < 0.001**	** < 0.001**	0.138
IOP (mmHg) (range)	16.76 ± 0.52 (7.5–24.0)	14.65 ± 0.38 (10.0–20.0)	14.07 ± 0.33 (10.0–21.0)	**0.001**	** < 0.001**	0.259
CCT (μm)	542.97 ± 4.52	531.54 ± 4.18	539.06 ± 5.06	0.063	0.564	0.252
BCVA	0.84 ± 0.03	0.95 ± 0.02	1.02 ± 0.02	**0.003**	** < 0.001**	**0.003**
ACD (mm)	2.48 ± 0.06	3.22 ± 0.08	3.52 ± 0.31	** < 0.001**	**0.001**	0.357
SAP MD (dB) (range)	−1.44 ± 0.24 (−5.73–1.17)	−1.55 ± 0.20 (−4.92– 1.65)	0.53 ± 0.08 (−1.82–1.96)	0.745	** < 0.001**	** < 0.001**
SAP PSD (dB) (range)	2.69 ± 0.20 (1.04–9.67)	3.10 ± 0.23 (1.39–10.55)	1.54 ± 0.04 (0.89–2.62)	0.172	** < 0.001**	** < 0.001**
SAP VFI (%) (range)	96.54 ± 0.56 (81–100)	96.30 ± 0.40 (82–100)	99.49 ± 0.08 (97–100)	0.721	** < 0.001**	** < 0.001**
Number of glaucoma medications	1.14 ± 0.13	0.82 ± 0.12	0	0.066	** < 0.001**	** < 0.001**

Significant values are in bold.

CCT = central corneal thickness; BCVA = best-corrected visual acuity; ACD = anterior chamber depth; SE = spherical equivalent; AL = axial length; IOP = intraocular pressure; SAP = standard automated perimetry; MD = mean deviation; PSD = pattern standard deviation; VFI = visual field index; PACG = primary angle closure glaucoma; NTG = normal tension glaucoma.

*Statistical significance was tested by generalized estimating equation (for continuous variables) or chi-square test (for categorical variables).

Table 2 shows the comparison of RNFL thickness and OCT-A metrics among the three groups. Whilst eyes in either glaucoma group have thinner RNFL thickness than the control eye (*P* < 0.001), there was no significant difference between the average RNFL thickness of PACG eyes and NTG eyes. NTG eyes had thinner RNFL thickness in the superotemporal and superonasal regions compared with PACG eyes (all *P* ≤ 0.038). Compared with normal eyes, eyes in either glaucoma group also showed lower global cpVD (both *P* ≤ 0.014) and lower cpFD (both *P* < 0.001). Comparing NTG eyes with PACG eyes, NTG eyes had a significantly lower global cpVD (52.369 ± 0.781% vs 55.389 ± 0.721%), lower VD at the superotemporal (53.410 ± 1.047% vs 57.094 ± 1.106%), inferotemporal (53.971 ± 1.129% vs 58.458 ± 0.909%), and inferonasal (48.879 ± 1.130% vs 54.946 ± 1.027%) regions (all *P* ≤ 0.031).

**Table 2:** Comparison of RNFL thickness and OCT-A metrics among PACG, NTG, and Controls.

Variables	PACG	NTG	Controls	*P* value		
				**PACG vs NTG**	**PACG vs Controls**	**NTG vs Controls**
**RNFL thickness (μm)**						
Average	88.00 ± 1.89	83.34 ± 1.62	102.45 ± 1.44	0.061	** < 0.001**	** < 0.001**
Superotemporal	120.93 ± 3.33	111.67 ± 2.97	140.79 ± 3.03	**0.038**	** < 0.001**	** < 0.001**
Temporal	69.48 ± 1.70	73.41 ± 1.82	86.31 ± 3.21	0.116	** < 0.001**	** < 0.001**
Inferotemporal	121.09 ± 3.98	112.99 ± 4.24	146.79 ± 4.32	0.163	** < 0.001**	** < 0.001**
Inferonasal	97.82 ± 2.99	92.48 ± 2.50	115.27 ± 3.08	0.171	** < 0.001**	** < 0.001**
Nasal	62.97 ± 1.90	59.92 ± 2.18	70.92 ± 2.72	0.287	**0.033**	**0.004**
Superonasal	98.21 ± 3.29	84.73 ± 3.28	111.24 ± 3.59	**0.007**	**0.007**	** < 0.001**
**cpVD (%)**						
Global	55.389 ± 0.721	52.369 ± 0.781	57.700 ± 0.599	**0.009**	**0.014**	** < 0.001**
Superotemporal	57.094 ± 1.106	53.410 ± 1.047	58.686 ± 0.828	**0.031**	0.249	** < 0.001**
Temporal	56.939 ± 0.977	56.920 ± 0.924	59.956 ± 0.755	0.989	**0.033**	**0.033**
Inferotemporal	58.458 ± 0.909	53.971 ± 1.129	60.817 ± 0.951	**0.004**	0.073	** < 0.001**
Inferonasal	54.946 ± 1.027	48.789 ± 1.130	56.658 ± 1.005	** < 0.001**	0.234	** < 0.001**
Nasal	51.643 ± 0.896	50.483 ± 1.130	54.547 ± 0.786	0.421	**0.030**	**0.009**
Superonasal	53.365 ± 0.903	50.457 ± 1.169	55.309 ± 0.768	0.098	0.101	**0.002**
**cpFD**	1.519 ± 0.004	1.516 ± 0.004	1.548 ± 0.003	0.523	** < 0.001**	** < 0.001**

Significant values are in bold.

Statistical significance was tested by generalized estimating equation with Sequential Bonferroni post-hoc test.

cpRNFL = circumpapillary retinal nerve fiber layer; cpVD = circumpapillary vessel density; cpFD = circumpapillary fractal dimension; PACG = primary angle closure glaucoma; NTG = normal tension glaucoma.

Univariate analyses of demographic (age, sex), ocular variables (SE, AL, IOP, CCT, BCVA, ACD), number of glaucoma medications, OCT-A quality score with average RNFL thickness and visual field parameters are summarized in Supplementary Table S2.

Table 3 shows the relationships of OCT-A metrics with average RNFL thickness in PACG, NTG, and normal controls. For the PACG and NTG group, after adjusting for age, sex, AL, IOP, number of glaucoma medications, and OCT-A image quality score, multivariable analyses showed that decreased cpVD ([PACG] β = −4.242; CI: −8.120, −0.363, and [NTG] β = −5.531; CI: −9.472, −1.590), and cpFD ([PACG] β = −8.894; CI: −11.925, −5.864, and [NTG]: β = −12.064; CI: −17.195, −6.932) were associated with decreased RNFL thickness (all *P* ≤ 0.032). In contrast, there was no significant association between OCT-A metrics and average RNFL thickness in normal eyes. The relationships of OCT-A metrics with the MD and VFI are shown in Table 4. In the multivariable analyses, decreased cpVD was significantly associated with decreased MD in NTG eyes (β = −0.707; CI: −1.090, −0.324; *P* < 0.001). However, in PACG eyes, there was no significant association between the OCT-A metrics and VF parameters. Univariate and multivariable analyses of diagnostic group, demographic variables (age, sex), ocular variables (SE, AL, IOP, CCT, BCVA, ACD, number of medications) and OCT-A quality score with OCT-A metrics (cpVD and cpFD) are summarized in the supplementary material (Supplementary Tables S3 and S4). In the multivariable regression model (Supplementary Table S4), which adjusting for age, sex, AL, IOP, BCVA, number of drugs, and OCT-A quality score, only the NTG group but not the PACG group had a significant association with cpVD and cpFD (*P* ≤ 0.001). We also compared the strength of associations of OCT-A metrics with average RNFL thickness between PACG and NTG (Table 5), revealing that there was a significantly stronger association between cpFD and average RNFL thickness in the NTG group compared with that in the PACG group (*P* = 0.028).

**Table 3:** Relationship of OCT-A metrics with average RNFL thickness in PACG, NTG, and Controls.

OCT-A metrics		Univariable model		Multivariable model	
		Average RNFL thickness, μm (95%CI)	*P* value	Average RNFL thickness, μm (95%CI)	*P* value
**PACG**					
cpVD (%)	Per SD decrease	−5.855 (−9.567, −2.143)	**0.002**	−4.242 (−8.120, −0.363)	**0.032**
cpFD	Per SD decrease	−9.297 (−11.682, −6.913)	** < 0.001**	−8.894 (−11.925, −5.864)	** < 0.001**
**NTG**					
cpVD (%)	Per SD decrease	−4.998 (−7.674, −2.322)	** < 0.001**	−5.531 (−9.472, −1.590)	**0.006**
cpFD	Per SD decrease	−9.831 (−15.901, −3.761)	**0.002**	−12.064 (−17.195, −6.932)	** < 0.001**
**Controls**					
cpVD (%)	Per SD decrease	1.918 (−0.684, 4.520)	0.148	2.221 (−0.197, 4.638)	0.072
cpFD	Per SD decrease	−0.646 (−3.284, 1.992)	0.631	−1.325 (−3.891, 1.241)	0.312

Significant values are in bold.

cpVD = circumpapillary vessel density; cpFD = circumpapillary fractal dimension; PACG = primary angle closure glaucoma; NTG = normal tension glaucoma; RNFL = retinal nerve fiber layer; CI = confidence interval; SD = standard deviation. Multivariable model adjusted for age, sex, spherical equivalent, intraocular pressure, number of glaucoma medications and OCT-A image quality score.

**Table 4:** Relationship of OCT-A metrics with visual field mean deviation and visual field index in PACG and NTG.

OCT-A metrics		Univariable model				Multivariable model			
		SAP MD, dB (95%CI)	*P* value	SAP VFI, % (95%CI)	*P* value	SAP MD, dB (95%CI)	*P* value	SAP VFI, % (95%CI)	*P* value
**PACG**									
cpVD (%)	Per SD decrease	−0.096 (−0.576, 0.385)	0.697	−0.714 (−1.715, 0.286)	0.162	−0.073 (−0.601, 0.455)	0.786	−1.304 (−3.070, 0.463)	0.148
cpFD	Per SD decrease	−0.345 (−0.914, 0.225)	0.236	−2.126 (−4.002, −0.250)	**0.026**	−0.490 (−1.713, 0.733)	0.433	−1.874 (−4.678, 0.931)	0.190
**NTG**									
cpVD (%)	Per SD decrease	−0.253 (−0.508, 0.002)	0.052	−0.334 (−0.724, 0.056)	0.093	−0.707 (−1.090, −0.324)	** < 0.001**	−0.338 (−1.100, 0.424)	0.385
cpFD	Per SD decrease	−0.221 (−0.600, 0.157)	0.252	−0.867 (−2.139, 0.406)	0.182	−0.032 (−1.106, 1.042)	0.953	−0.424 (−1.621, 0.774)	0.488
**Controls**									
cpVD (%)	Per SD decrease	0.010 (−0.162, 0.181)	0.913	0.036 (−0.151, 0.223)	0.706	−0.002 (−0.172, 0.167)	0.977	0.015 (−0.173, 0.202)	0.880
cpFD	Per SD decrease	0.126 (−0.076, 0.329)	0.222	−0.098 (−0.331, 0.135)	0.409	0.132 (−0.088, 0.351)	0.239	−0.168 (−0.405, 0.069)	0.165

Significant values are in bold.

cpVD = circumpapillary vessel density; cpFD = circumpapillary fractal dimension; PACG = primary angle closure glaucoma; NTG = normal tension glaucoma; SAP = standard automated perimetry; MD = mean deviation; PSD = pattern standard deviation; VFI = visual field index; CI = confidence interval; SD = standard deviation. Multivariable model adjusted for age, sex, spherical equivalent, intraocular pressure, number of glaucoma medications and OCT-A image quality score.

**Table 5:** Comparison of regression coefficients of OCT-A metrics with average RNFL thickness between PACG and NTG.

OCT-A metrics	Univariable model	Multivariable model
	β (95% CI)	β (95% CI)
cpVD (%)		
PACG	−5.855 (−9.567, −2.143)*	−4.242 (−8.120, −0.363)*
NTG	−4.998 (−7.674, −2.322)**	−5.531 (−9.472, −1.590)*
*P* value	0.449	0.658
cpFD		
PACG	−9.297 (−11.682, −6.913)**	−8.894 (−11.925, −5.864)**
NTG	−9.831 (−15.901, −3.761)*	−12.064 (−17.195, −6.932)**
***P* value**	**0.002**	**0.028**

Significant values are in bold.

cpVD = circumpapillary vessel density; cpFD = circumpapillary fractal dimension; PACG = primary angle closure glaucoma; NTG = normal tension glaucoma; RNFL = retinal nerve fiber layer; β = regression coefficient; CI = confidence interval.

*Denotes *P* < 0.05; **denotes *P* < 0.001; OCT-A metrics were analyzed as per SD decrease. Multivariable regressions were adjusted for age, sex, spherical equivalent, intraocular pressure, number of glaucoma medications and OCT-A image quality score.

## Discussion

Our findings of reduced cpVD and cpFD of glaucomatous eyes compared with age-matched normal controls were in concordance with previous studies that showed reduction of the microvascular density of the optic disc in glaucoma patients^11, 29–32^. Rao et al.^30^ reported that VD in optic disc region in PACG eyes was significantly lower than control eyes, whereas VD in primary angle closure (PAC) with high IOP and thinner superotemporal peripapillary RNFL thickness was similar to that of the controls. They suggested that high IOP affects the RNFL measurements earlier than VD in PACG. Scripsema et al.^11^ found that both POAG and NTG patients demonstrated decreased perfused capillary density compared to normal subjects, with POAG patients having a lower perfused capillary density than NTG patients. They attributed the latter finding to be possibly related to medication effect (POAG patients using larger number of drops) and/or the different pathophysiological processes of NTG and HTG.

To our knowledge, this is the first study to compare the peripapillary microvasculature in early PACG and early NTG eyes using OCT-A. We utilized OCT-A to compare the peripapillary microvasculature of two subtypes of early glaucoma which have likely different pathogenic mechanisms—NTG that is less IOP dependent and has possibly a stronger vascular pathogenic component^5, 33^, and PACG that has likely a predominantly IOP-dependent mechanism^34^. This allowed a “snapshot” comparison of the detailed microvasculature between NTG and PACG at the early stage of the disease. Our results showed a significantly reduced global cpVD in NTG eyes compared with PACG eyes, despite the comparable RNFL thickness and disease severity. Our study also reported that early NTG had a more evenly distributed reduction of cpVD in each sector, whereas early PACG had a significantly lower cpVD only in the temporal and nasal sectors compared with normal controls. Furthermore, NTG eyes showed a significantly lower cpVD in the inferotemporal and inferonasal sectors compared with PACG eyes despite a similar RNFL thickness in these sectors, suggesting a different pattern of ocular perfusion change of the two glaucoma subtypes. We also identified a significant association between the OCT-A metrics and RNFL thickness in both glaucoma groups, with a stronger relationship between the cpFD and RNFL thickness in the NTG group compared with the PACG group. The findings might reflect a more specific and early reduction of microvascular perfusion at the peripapilliary region of NTG eyes at early stage glaucoma. Such a difference was not observed in other studies that compared patients with PACG and POAG (involved both NTG and HTG)^35, 36^.

Given the similar disease severity and the number of medications used, the difference in cpVD between NTG and PACG eyes could be related to the different pathogenic mechanisms of the two glaucoma subtypes. For NTG at the early stage, the impairment of vascular autoregulation—postulated to be an important risk factor for disease progression in NTG^5, 6, 37–39^—could lead to reduced blood flow but has yet to cause dysfunction, death, or atrophy of the RGC, as well as the consequential RNFL thinning. Therefore, in NTG eyes, there was a reduction of peripapillary microvascular perfusion prior to RGCs loss, and a further delay for the development of a detectable RNFL thinning after the RGCs’ loss. The latter is supported by in vivo study that showed an initially faster decline of RGC soma counts compared with RNFL thickness following optic nerve injury in animal model^40^. Our findings of the stronger association between cpFD and RNFL thickness in NTG eyes compared with PACG eyes, as well as the association between decrease in cpVD and decrease MD of VF in NTG (which was not observed in PACG eyes), may provide a modest but concordant support of this theory. Further in vivo studies are needed to validate the role of microvasculature in the pathophysiology in NTG. In PACG eyes, the loss of RGCs was possibly mainly due to elevation of IOP that occurred prior to IOP lowering treatments (including lens extraction and/or laser iridotomy) and the reduction of the OCT-A metrics could be a secondary consequence of RGCs loss; this echoed with the suggestion of Rao et al. that high IOP affects the RNFL measurements earlier than VD in PACG^30^. The atrophy of RGCs may lead to a reduced demand of blood supply and blood flow that was reflected as a reduction in cpVD. However, this secondary reduction of cpVD, unlike NTG eyes at a similar stage of disease severity, was not extensive enough to a degree that would cause further RGCs loss. Hence, this possible difference in the pathogenic algorithm of NTG and PACG might lead to the differences in cpVD measurement between the two subgroups, despite similar RNFL thickness. A longitudinal study with larger number of patients may, in the future, verify the causal relationship of vascular-RNFL thickness in these two subtypes of glaucoma. This is not only important in terms of understanding the pathophysiology of the disease, it is also clinically implicative if OCT-A is to be utilized as a diagnostic and monitoring tool in glaucoma management.

In the multivariable analyses, we found a significant association between OCT-A metrics and RNFL thickness in both glaucoma subtypes but only an association between cpVD and MD in the NTG group (Table 4). This differed from previous studies that reported a stronger association between decreased cpVD with the severity of VF damage, compared with the association between RNFL thickness and VF function in PACG^35^ and POAG eyes^14, 35^. However, our results were expected because we only included glaucoma patients with mild severity with the MD score of better than −6.0 D. Identifying an association between OCT-A metrics within a narrow range of VF parameters (MD of −5.73 to 1.65 dB and VFI of 81 to 100% in the glaucoma groups) is understandably difficult. Furthermore, the association between microvasculature with functional change may not be strong in these patients with early stage disease that have minimal functional loss. Indeed, a study by Shin et al. showed that whilst there was a significant relationship between VD and VF function in moderate-to-advance POAG regardless of location, the relationship of VD and VF function was only significant in the superotemporal and inferotemporal regions for early stage POAG^41^. Although both MD and VFI reflect the severity of visual field loss, our results showed that only MD but not VFI was significantly association with cpVD in NTG eyes (Table 4). This could be explained by VFI underestimating the vasculature change in the early disease stage. This deduction is supported by a previous study^42^ showing that VFI demonstrated a weak correlation with the structural measures compared with MD.

The strength of our study was the inclusion of early NTG and PACG patients—that have similar age, disease severity, RNFL thickness and number of medications used—for comparison. This could better reflect the role of microvasculature in the glaucomatous pathogenic mechanisms at the early stage of the disease. We also used an objective, automated MATLAB program to quantitatively measure retinal microvasculature. Limitations of this study included a cross-sectional study design, a relatively small sample size, not taking the macular region into the analysis with visual field, as well as not taking systemic diseases into consideration (e.g. obstructive sleep apnea, hypertension, ocular perfusion pressure). Further studies are essentially needed to confirm our results with consideration of the potential confounding effect of systemic vascular factors. We have limited the range of refractive errors (+ 3.0 to − 3.0 D) in an attempt to avoid inclusion of eyes with extreme AL. We acknowledge that the AL of PACG eyes were statistically shorter than either the NTG eyes or control eyes in the current study (Table 1). However, the difference in AL between the PACG and NTG group was reasonable in the clinical point of view (22.43 ± 0.12 D vs. 24.60 ± 0.17 D; *P* < 0.001). Nonetheless, our findings provide the basis for future longitudinal study that may reveal the causal relationship between retinal microvasculature change and RNFL thickness change.

In summary, the cpVD was significantly lower in early NTG eyes when compared to early PACG eyes, despite similar RNFL thickness and VF parameters. Reductions in cpVD and cpFD were associated with average RNFL thickness thinning in both NTG and PACG eyes. Longitudinal study may verify the differences of microvasculature change in different glaucoma subtypes, improve our understanding of the pathogenic mechanisms of the diseases, and also establish a role for OCT-A in the management of glaucoma.

## Supplementary Information

Supplementary Information.
