# Supplementary material for: Comparison of optical coherence tomography angiography metrics in primary angle-closure glaucoma and normal-tension glaucoma
Source: Sci Rep. 2021 Nov 30;11:23136. doi: 10.1038/s41598-021-02296-x (PMC8632996; doi:10.1038/s41598-021-02296-x)
Supplement: Supplementary file 1 — Supplementary Information. [file 41598_2021_2296_MOESM1_ESM.docx]

**Supplementary Table 1.** Inter- and Intra-observer Reliability on measurement of OCT-A metrics.

|  | **Intergrader Reliability** | | | | **Intragrader Reliability** | | | |
| --- | --- | --- | --- | --- | --- | --- | --- | --- |
|  | **ICC** | **95%CI lower bound** | **95%CI upper bound** | **ICC** | | **95%CI lower bound** | **95%CI upper bound** |  |
| **cpVD (%)** |  |  |  |  | |  |  |  |
| Global | 0.991 | 0.978 | 0.996 | 0.996 | | 0.991 | 0.998 |  |
| Superotemporal | 0.988 | 0.975 | 0.994 | 0.993 | | 0.986 | 0.997 |  |
| Temporal | 0.982 | 0.943 | 0.993 | 0.987 | | 0.974 | 0.994 |  |
| Inferotemporal | 0.978 | 0.944 | 0.991 | 0.992 | | 0.983 | 0.996 |  |
| Inferonasal | 0.970 | 0.925 | 0.987 | 0.989 | | 0.976 | 0.995 |  |
| Nasal | 0.996 | 0.992 | 0.998 | 0.996 | | 0.992 | 0.998 |  |
| Superonasal | 0.994 | 0.987 | 0.997 | 0.987 | | 0.972 | 0.994 |  |
| **cpFD** | 0.968 | 0.934 | 0.985 | 0.990 | | 0.979 | 0.995 |  |

CI = confidence interval; ICC=intraclass correlation coefficient; cpVD = circumpapillary vessel density; cpFD = circumpapillary fractal dimension.

**Supplementary Table 2**: Univariate regression analyses of variables with average RNFL thickness, SAP MD and SAP VFI.

| **Variables** |  | **Average RNFL thickness** | | **SAP MD** | | **SAP VFI** | |
| --- | --- | --- | --- | --- | --- | --- | --- |
|  |  | **β (95% CI)** | ***P* value** | **β (95% CI)** | ***P* value** | **β (95% CI)** | ***P* value** |
| Age | Per SD increase | -2.848  (-4.932, -0.765) | **0.007** | -0.160  (-0.417, 0.098) | 0.224 | -0.144  (-0.634, 0.346) | 0.565 |
| Gender | Female : male | 1.549  (-3.260, 6.357) | 0.528 | -0.505  (-1.044, 0.034) | 0.066 | -0.785  (-1.740, 0.170) | 0.107 |
| SE | Per SD increase | 1.124  (-0.968, 3.216) | 0.292 | 0.030  (-0.236, 0.296) | 0.824 | 0.532  (-0.050, 1.114) | 0.073 |
| AL | Per SD increase | -0.357  (-2.607, 1.894) | 0.756 | 0.186  (-0.084, 0.456) | 0.176 | -0.084  (-0.636, 0.468) | 0.765 |
| IOP | Per SD increase | -4.025  (-6.206, -1.844) | **<0.001** | -0.168  (-0.488, 0.153) | 0.306 | 0.008  (-0.697, 0.712) | 0.983 |
| CCT | Per SD increase | 1.425  (-0.994, 3.845) | 0.248 | 0.175  (-0.063, 0.414) | 0.150 | 0.361  (-0.040, 0.761) | 0.077 |
| BCVA | Per SD increase | 2.280  (-0.040, 4.600) | 0.054 | 0.181  (-0.055, 0.418) | 0.132 | 0.134  (-0.315, 0.582) | 0.559 |
| ACD | Per SD increase | -0.239  (-0.791, 0.314) | 0.397 | 0.050  (-0.050, 0.151) | 0.323 | -0.050  (-0.146, 0.045) | 0.300 |
| Number of glaucoma medications | Per SD increase | -6.819  (-8.830, -4.808) | **<0.001** | -0.529  (-0.738, -0.319) | **<0.001** | -0.690  (-1.160, -0.220) | **0.004** |
| OCT-A quality score | Per SD increase | -3.237  (-5.032, -1.442) | **<0.001** | -0.156  (-0.339, 0.027) | 0.095 | -0.171  (-0.479, 0.137) | 0.276 |

SE = spherical equivalent; AL = axial length; IOP = intraocular pressure; CCT = central corneal thickness; BCVA = best-corrected visual acuity; ACD = anterior chamber depth; RNFL = retinal nerve fiber layer; SAP = standard automated perimetry; MD = mean deviation; VFI = visual field index.

**Supplementary** **Table 3**. Univariate regression of variables with cpVD and cpFD..

| **Variables** |  | **cpVD** | | **cpFD** | |
| --- | --- | --- | --- | --- | --- |
|  |  | **β (95% CI)** | ***P* value** | **β (95% CI)** | ***P* value** |
| Age | Per SD increase | -0.628 (-1.551, 0.295) | 0.182 | -0.006 (-0.010, -0.001) | **0.011** |
| Gender | Female : Male | 1.665 (-0.202, 3.531) | 0.080 | 0.005 (-0.004, 0.015) | 0.261 |
| SE | Per SD increase | -0.245 (-1.024, 0.534) | 0.537 | -0.007 (-0.011, -0.004) | **<0.001** |
| AL | Per SD increase | -0.212 (-1.042, 0.618) | 0.617 | 0.007 (0.004, 0.011) | **<0.001** |
| IOP | Per SD increase | -0.421 (-1.269, 0.426) | 0.330 | -0.006 (-0.010, -0.001) | **0.018** |
| CCT | Per SD increase | -0.150 (-1.061, 0.762) | 0.748 | 0.001 (-0.003, 0.006) | 0.633 |
| BCVA | Per SD increase | 0.393 (-0.411, 1.197) | 0.338 | 0.006 (0.001, 0.010) | **0.010** |
| ACD | Per SD increase | -0.113 (-0.413, 0.188) | 0.462 | 0.000 (-0.001, 0.002) | 0.434 |
| Number of glaucoma medications | Per SD increase | -1.470 (-2.267, -0.672) | **<0.001** | -0.011 (-0.015, -0.007) | **<0.001** |
| OCT-A quality score | Per SD increase | -0.843 (-1.648, -0.038) | **0.040** | -0.004 (-0.007, 0.000) | **0.028** |
| PACG vs. Controls | | -2.311 (-4.148, -0.474) | **0.014** | -0.029 (-0.038, -0.021) | **<0.001** |
| NTG vs. Controls | | -5.331 (-7.259, -3.403) | **<0.001** | -0.033 (-0.042, -0.023) | **<0.001** |

SE = spherical equivalent; AL = axial length; IOP = intraocular pressure; CCT = central corneal thickness; BCVA = best-corrected visual acuity; ACD = anterior chamber depth; PACG = primary angle closure glaucoma; NTG = normal tension glaucoma. cpVD = circumpapillary vessel density; cpFD = circumpapillary fractal dimension.

**Supplementary** **Table 4**. Multivariable regression of variables with cpVD and cpFD.

| **Variables** |  | **cpVD** | | **cpFD** | |
| --- | --- | --- | --- | --- | --- |
|  |  | **β (95% CI)** | ***P* value** | **β (95% CI)** | ***P* value** |
| Age | Per SD increase | -0.270 (-1.187, 0.646) | 0.563 | -0.003 (-0.007, 0.001) | 0.176 |
| Gender | Female : Male | 1.013 (-0.902, 2.927) | 0.300 | 0.005 (-0.004, 0.013) | 0.315 |
| AL | Per SD increase | -0.395 (-1.413, 0.622) | 0.446 | 0.006 (0.001, 0.012) | **0.026** |
| IOP | Per SD increase | 0.093 (-0.754, 0.939) | 0.830 | -0.004 (-0.009, 0.001) | 0.095 |
| BCVA | Per SD increase | -0.275 (-1.336, 0.785) | 0.611 | 0.001 (-0.005, 0.007) | 0.692 |
| Number of glaucoma medications | Per SD increase | -1.183 (-2.137, -0.228) | **0.015** | -0.005 (-0.010, -0.001) | **0.019** |
| OCT-A quality score | Per SD increase | 0.011 (-0.798, 0.820) | 0.978 | 0.002 (-0.004, 0.009) | 0.451 |
| PACG vs. Controls |  | -2.740 (-5.574, 0.093) | 0.058 | -0.010 (-0.022, 0.003) | 0.124 |
| NTG vs. Controls |  | -4.074 (-6.383, -1.765) | **0.001** | -0.030 (-0.042, -0.018) | **<0.001** |

AL = axial length; IOP = intraocular pressure; BCVA = best-corrected visual acuity; PACG = primary angle closure glaucoma; NTG = normal tension glaucoma.
